# *Salmonella enterica* Serotype Typhi with Nonclassical Quinolone Resistance Phenotype

**DOI:** 10.3201/eid1706.101242

**Published:** 2011-06

**Authors:** Marie Accou-Demartin, Valérie Gaborieau, Yajun Song, Philippe Roumagnac, Bruno Marchou, Mark Achtman, François-Xavier Weill

**Affiliations:** Author affiliations: Institut Pasteur, Paris, France (M. Accou-Demartin, F.-X. Weill);; Hôpital Purpan, Toulouse, France (V. Gaborieau, B. Marchou);; University College Cork, Cork, Ireland (Y. Song, M. Achtman);; Centre de Coopération International en Recherche Agronomique pour le Développement, Montpellier, France (P. Roumagnac)

**Keywords:** Salmonella, typhoid fever, ciprofloxacin, treatment failure, gyrB, bacteria, antimicrobial resistance, expedited, dispatch

## Abstract

We report *Salmonella enterica* serotype Typhi strains with a nonclassical quinolone resistance phenotype (i.e., decreased susceptibility to ciprofloxacin but with susceptibility to nalidixic acid) associated with a nonsynonymous mutation at codon 464 of the *gyrB* gene. These strains, not detected by the nalidixic acid disk screening test, can result in fluoroquinolone treatment failure.

Typhoid fever caused by *Salmonella enterica* serotype Typhi (hereafter referred to as *Salmonella* Typhi) remains a major health problem in the developing world (*1*). Treatment with appropriate antimicrobial drugs has become hampered by gradual plasmid-mediated resistance to ampicillin, chloramphenicol, and cotrimoxazole, particularly in southern and Southeast Asia (*2*). Consequently, since the early 1990s, fluoroquinolones (such as ofloxacin and ciprofloxacin [Cip]) have been widely used. However, multidrug-resistant *Salmonella* Typhi isolates that are also resistant to nalidixic acid (Nal^R^) (MIC >256 μg/mL) and show decreased susceptibility to Cip (Cip^DS^) (MIC range, 0.125 μg/mL–1 μg/mL) have emerged and become endemic on the Indian subcontinent and in Southeast Asia (*3**–**5*). This resistance to quinolones was caused by amino acid substitutions in the quinolone resistance–determining region (QRDR) of the DNA gyrase subunit *gyrA*, a key target of quinolones. Because these Nal^R^–Cip^DS^ *Salmonella* Typhi strains have been associated with slower clinical responses to fluoroquinolones and treatment failures, clinical laboratories should attempt to identify these isolates (*3**,**6**,**7*). However, despite the accumulation of clinical, microbiologic, and pharmacokinetic–pharmacodynamic studies suggesting a resistance breakpoint of >0.125 μg/mL for ciprofloxacin, the clinical breakpoints published by the Clinical and Laboratory Standards Institute (CLSI) (susceptibility <1 μg/mL, resistance >4 μg/mL) and those from the antibiogram committee of the French Society for Microbiology (susceptibility <0.5 μg/mL, resistance >1 μg/mL) (www.sfm.asso.fr/nouv/general.php?pa=2) have not been reevaluated (*6**–**9*). Use of these standard breakpoints has probably resulted in the underreporting of Cip^DS^ *Salmonella* Typhi strains. The Nal^R^ screening test has been proposed as an alternative since the mid–1990s and recommended since 2004 by CLSI and 2010 by the French Society for Microbiology (*3**,**7*). This screening test is based on the fact that Cip^DS^ *Salmonella* Typhi isolates with nonsynonymous (NS) mutations in codons 83 or 87 of *gyrA* are uniformly Nal^R^. However, recent reports have indicated that this approach cannot identify the newly described *Salmonella* Typhi isolates that are Nal susceptible (Nal^S^)–Cip^DS^ for which mechanisms of resistance are not linked to mutations in *gyrA* (*7**,**10**,**11*). Recently, NS mutations in codons 464 (Ser to Phe) and 466 (Glu to Asp) of *gyrB* were found in 7 Nal^S^–Cip^DS^ *Salmonella* Typhi isolates (*12*). We present data on the occurrence and characterization of the resistance mechanisms of Nal^S^–Cip^DS^ isolates in 685 *Salmonella* Typhi isolates of the French National Reference Center for *Salmonella* (FNRC-Salm).

## The Study

**Figure 1:**
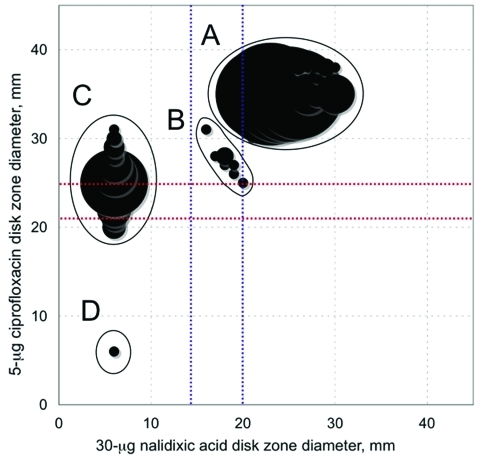
Scattergrams for 685 *Salmonella enterica* serotype Typhi isolates correlating the zone diameters around the 5-μg ciprofloxacin disk with those of the 30-μg nalidixic acid disk. Circle sizes are proportional to the number of isolates. Red lines indicate the respective antibiogram committee of the French Society for Microbiology (CA-SFM) breakpoints for ciprofloxacin (susceptible [S] >25; resistant [R] <22 mm). Blue lines indicate the respective CA-SFM breakpoints for nalidixic acid (S >20 and R <15 mm).

In France, laboratory surveillance of typhoid fever infections is performed by the FNRC-Salm through its network of ≈1,500 hospital and private clinical laboratories. Almost all *Salmonella* Typhi isolates in France are referred to the FNRC-Salm, and almost all are acquired abroad, mainly in Africa and Asia. Until 2009, Cip^DS^ *Salmonella* Typhi was monitored with the 30-μg Nal screening test. A total of 685 *Salmonella* Typhi isolates collected during 1997–2009 were reanalyzed to identify Nal^S^–Cip^DS^ *Salmonella* Typhi isolates. The scattergram correlating the zone diameters around the 5-μg ciprofloxacin disk with those of the 30-μg Nal disk showed 4 subpopulations, which were labeled A (554 isolates), B (11 isolates), C (119 isolates), and D (1 isolate) (Figure 1). The characteristics of these populations are shown in Table 1 and Table 2. The QRDRs of *gyrA*, *gyrB*, *parC,* and *parE* genes were studied on 133 isolates selected to represent diversity in terms of year of isolation, geographic origin, and MICs. To analyze the isolate characteristics, we used the following approaches: sequencing (*5*), denaturing high performance liquid chromatography (*4*), and Luminex-based genotyping assays (*12*). QRDR DNA sequences were compared with those of *Salmonella* Typhi strain Ty2 (GenBank accession no. AE014613). In subpopulation A, 75 isolates had wild-type QRDR sequences, whereas 2 isolates had a *gyrB* mutation at codon 465 leading to amino acid substitution Gln to Leu. Their Nal MICs were 2 and 4 μg/mL, respectively, and those of Cip were 0.04 μg/mL and 0.08 μg/mL, respectively. Notably, both isolates were acquired in Mexico during 1998 and 2009, respectively. In subpopulation C, the lowest MIC values for Cip (0.06 μg/mL) were associated with a mutation at codon 87 of the *gyrA* gene, whereas MICs did not increase with the additional mutation in the *parE* gene. Subpopulation D consisted of 1 isolate, highly resistant to ciprofloxacin, which was acquired by a traveler in India in 2004. This isolate contained 2 NS mutations in the *gyrA* gene and 1 in the *parC* gene.

**Table 1:** Characteristics of the 674 *Salmonella enterica* serovar Typhi isolates belonging to subpopulations A, C, and D, France, 2007–2009*

Subpopulation	No. isolates	Nal MICs,† μg/mL				Cip MICs,† μg/mL			QRDR mutation (no./no. tested)
MIC_50_	MIC_90_	Range	MIC_50_	MIC_90_	Range
A	554	4	4	1–8		0.008	0.025	0.002–0.08	WT (75/77)
									*gyrB* Leu465 (2/77)
C	119	>256	>256	128–>256		0.25	0.5	0.06–0.5	*gyrA* Phe83 (24/44)
									*gyrA* Tyr83 (12/44)
									*gyrA* Asn87 (4/44)
									*gyrA* Gly87 (2/44)
									*gyrA* Phe83 and *parE* Asn420 (2/44)
D	1			>256				8	*gyrA* Phe83, *gyrA* Asn87, and *parC* Ile80

*Nal, nalidixic acid; Cip, ciprofloxacin; QRDR, quinolone resistance–determining region of the *gyrA*, *gyrB*, *parC,* and *parE* genes; WT, wild type*.* †MICs of Nal and Cip were determined by Etest strips. MIC_50_, 50% below; MIC_90_, 90% below.

_._

**Table 2:** Characteristics of the 11 *Salmonella enterica* serovar Typhi isolates belonging to subpopulation B, France, 2007–2009

Isolate	Year	Geographic origin	Antimicrobial drug resistance type	Disk diffusion, mm			MIC, μg/mL		*gyrB*	Haplotype	PFGE
				Nal	Cip		Nal	Cip			
97-5123	1997	Unknown	Cip^DS^	18 [I]	28 [S]		8 [S/S]	0.125 [S/S]	Tyr464	Non-H58	X8
02-2759	2002	India	Cip^DS^	19 [I]	26 [S]		4 [S/S]	0.125 [S/S]	Phe464	H58	X2
05-1578	2005	India	Pansusceptible	18 [I]	28 [S]		8 [S/S]	0.047 [S/S]	Asp466	Non-H58	X6
05-2556	2005	India	Cip^DS^	17 [I]	31 [S]		16 [I/S]	0.19 [S/S]	Phe464	Non-H58	X7
05-9141	2005	India	Cip^DS^	17 [I]	28 [S]		12 [I/S]	0.125 [S/S]	Tyr464	Non-H58	X3
06-426	2006	India	Cip^DS^	20 [S]	25 [S]		8 [S/S]	0.125 [S/S]	Tyr464	Non-H58	X3
07-6086	2007	Tunisia	Pansusceptible	16 [I]	31 [S]		16 [I/S]	0.047 [S/S]	WT	ND	ND
08-7675†	2008	India	ASCSulTmpSXTCip^DS^	18 [I]	28 [S]		8 [S/S]	0.125 [S/S]	Phe464	H58	X1
09-1986†	2008	India	ASCSulTmpSXTCip^DS^	18 [I]	27 [S]		8 [S/S]	0.125 [S/S]	Phe464	ND	X1
09-0350	2009	Unknown	Cip^DS^	19 [I]	27 [S]		8 [S/S]	0.125 [S/S]	Phe464	Non-H58	X5
09-2317	2009	French Guyana	Pansusceptible	19 [I]	32 [S]		8 [S/S]	0.032 [S/S]	Glu468	Non-H58	X4

*PFGE, pulsed-field gel electrophoresis; Nal, nalidixic acid; Cip, ciprofloxacin; WT, wild type; ND, not determined; A, ampicillin; S, streptomycin, C, chloramphenicol; Su, sulfamethoxazole; Tmp, trimethoprim; SXT, cotrimoxazole; Cip^DS^, decreased susceptibility to ciprofloxacin. Disk diffusion test was performed and interpreted ([S], susceptible; [I], intermediate) following recommendations of antibiogram committee of the French Society for Microbiology. MICs were determined by Etest strips, and categorization was made according to the French Society for Microbiology and Clinical and Laboratory Standards Institute. †Previously described same patient (*13*).

**Figure 2:**
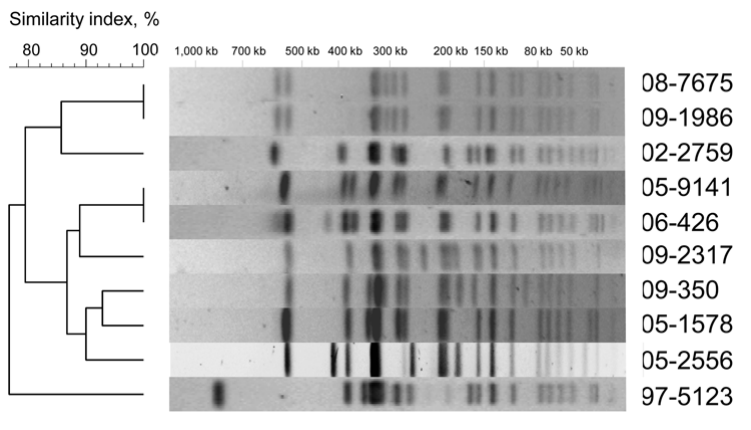
*Xba*I pulsed-field gel electrophoresis (PFGE) profiles obtained from 10 *Salmonella enterica* serotype Typhi isolates belonging to subpopulation B. The dendrograms generated by BioNumerics version 3.5 software (Applied Maths, Sint-Martens-Latem, Belgium) show the results of cluster analysis on the basis of PFGE fingerprinting. Similarity analysis was performed by using the Dice coefficient, and clustering was done by using the unweighted pair-group method with arithmetic averages.

Eleven isolates of subpopulation B were categorized as susceptible to Nal by determining MICs and by using CLSI breakpoints (susceptibility, <16 μg/mL; resistance, >32 μg/mL). Of the 11 isolates, 8 (from 7 patients) had a ciprofloxacin MIC >0.125 μg/mL and were thus classified as Cip^DS^ isolates. We were able to review the medical records of 2 patients infected with a Nal^S^–Cip^DS^ isolate. One patient (isolates 08-7675 and 09-1986) relapsed 15 days after completion of the treatment (oral ofloxacin, 200 mg 2×/d for 8 days) (*13*). The second patient (isolate 05-2556) was treated with extended-spectrum cephalosporins, and no fluoroquinolones. Regarding the resistance mechanisms the plasmid-mediated quinolone resistance–conferring genes *qnr (qnrA, B, S, D), qepA,* and *aac(6′)-Ib-cr* were not detected by PCR (*5**,**14*). The QRDRs of *gyrA*, *parC*, and *parE* genes were of a wild type, whereas an NS mutation was found in *gyrB* for all but 1 isolate. However, only the 8 isolates with mutations at codon 464 were Nal^S^–Cip^DS^. To assess whether these isolates were genetically related, haplotyping (*4*) and *Xba*I-pulsed-field gel electrophoresis (PFGE) subtyping (*5*) were performed. On the strength of the results, we concluded that the *gyrB* mutation was acquired independently by strains belonging to different PFGE types (Figure 2). According to a newly developed single nucleotide polymorphism assay (Y.S.), 2 of these strains belong to the current emerging H58 Asian population (*4*), whereas the others do not (Table 2). In our study, the Nal^S^–Cip^DS^ isolates with *gyrB* mutations at codon 464 were most often non–multidrug-resistant and acquired mainly in India. Our first Nal^S^–Cip^DS^ isolate was isolated 13 years ago, and since is rare (prevalence ≈1%.). Although Cooke et al. (*10*) did not characterize isolates for their resistance mechanisms, they reported that Nal^S^–Cip^DS^ represented 11.6% (49/421) of *Salmonella* Typhi isolated in England, Scotland, and Wales during 1999–2003, while Lynch et al. (*11*) reported that such isolates were 4.6% (36/770) of *Salmonella* Typhi isolates identified in the United States during 1999–2006. Epidemiologic data were available for 39 isolates in the British study, 18 of which were acquired in India, 8 in Pakistan, and 4 in Bangladesh (*10*). The 10-fold difference in the prevalence observed between our study and that of Cooke et al. are probably related to the historical links and the subsequent population flow between the United Kingdom and the Indian subcontinent.

## Conclusions

Nal^S^–Cip^DS^ *Salmonella* Typhi isolates originating from Asia comprise ≈1% of *Salmonella* Typhi isolates in France but are more prevalent in the United States and the United Kingdom. The NS *gyrB* mutation at codon 464 was found exclusively in Nal^S^–Cip^DS^ isolates; however, the effects of this mutation need to be formally demonstrated by site-directed mutagenesis. Furthermore, the involvement of an efflux system, such as AcrAB-TolC and OqxA, or the *qnrC* gene, have not been investigated and cannot be excluded.

Whatever the molecular mechanism of resistance of such strains, the main concern is detection of such isolates in clinical practice to prevent fluoroquinolone treatment failures. Consequently, the Nal^R^ screening test should no longer be recommended and ciprofloxacin drug MICs should be determined for all *Salmonella* Typhi isolates instead. There is also a clear need to reevaluate the clinical breakpoints for this pathogen.
